# Supplementary material for: A micro-scale simulation of red blood cell passage through symmetric and asymmetric bifurcated vessels
Source: Sci Rep. 2016 Feb 2;6:20262. doi: 10.1038/srep20262 (PMC4735796; doi:10.1038/srep20262)
Supplement: Supplementary Information [file srep20262-s1.pdf]

A micro-scale simulation of red blood cell passage through symmetric and asymmetric bifurcated vessels

Tong Wang[1,*], Uwitije Rongin[1], and Zhongwen Xing[2,†]

[1]Department of Mathematics, Nanjing University of Aeronautics and Astronautics, Nanjing 210016, China
[2]Department of Materials Science and Engineering, Nanjing University, Nanjing 210093, China
[*]twang@nuaa.edu.cn
[†]zwxing@nju.edu.cn

Supplementary Information

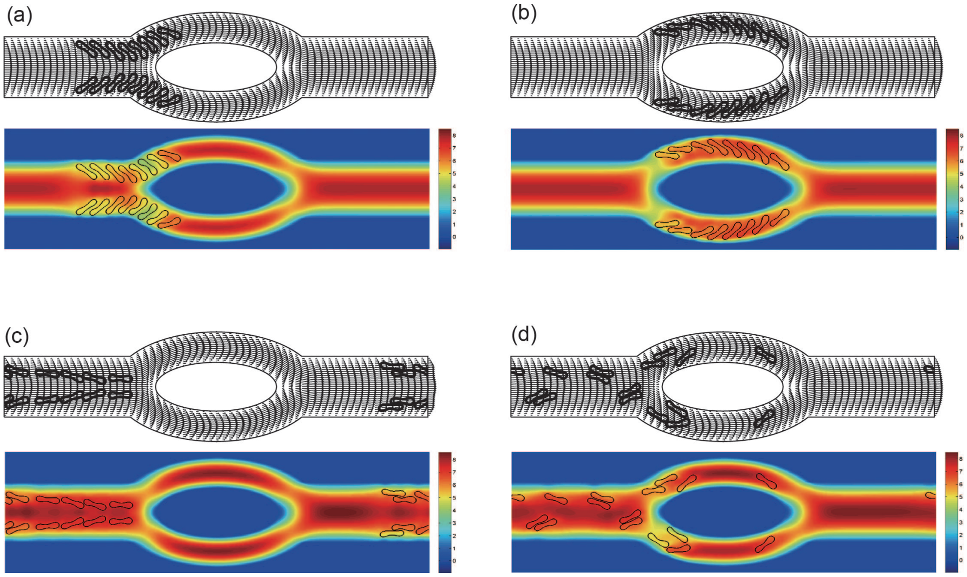

Supplementary Figure S 1: (Motion of two files of 16 red blood cells (Hct=6.4 %) in the symmetric bifurcated microchannel at time instants (a) t = 0.62 ms, (b) t = 1.12 ms, (c) t = 2.40 ms, and (d) t = 5.00 ms. Velocity vectors (upper panels) and axial velocity magnitude contours (cm/s) (lower panels) are presented. The reduced area s^*=0.481. The spring constant of the cell membrane was $k_l = k_b = 3.0 \times 10^{-13}$ Nm.

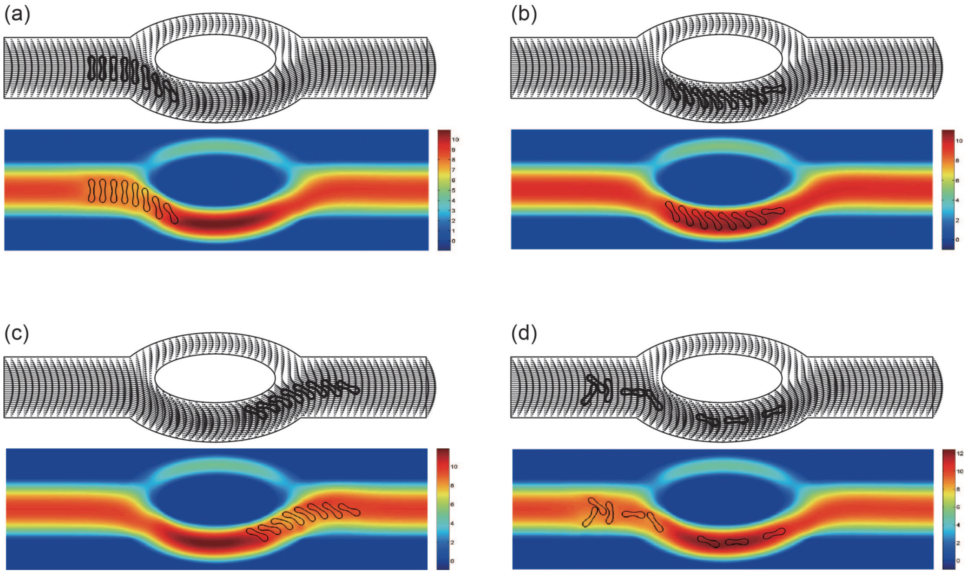

Supplementary Figure S 2: Motion of a file of 8 red blood cells (Hct=3.2 %) in the asymmetric bifurcated microchannel at time instants (a) t = 0.48 ms, (b) t = 0.80 ms, (c) t = 1.10 ms, and (d) t = 5.00 ms. Velocity vectors (upper panels) and axial velocity magnitude contours (cm/s) (lower panels) are presented. The reduced area s^*=0.481. The spring constant of the cell membrane was $k_l = k_b = 3.0 \times 10^{-13}$ Nm.

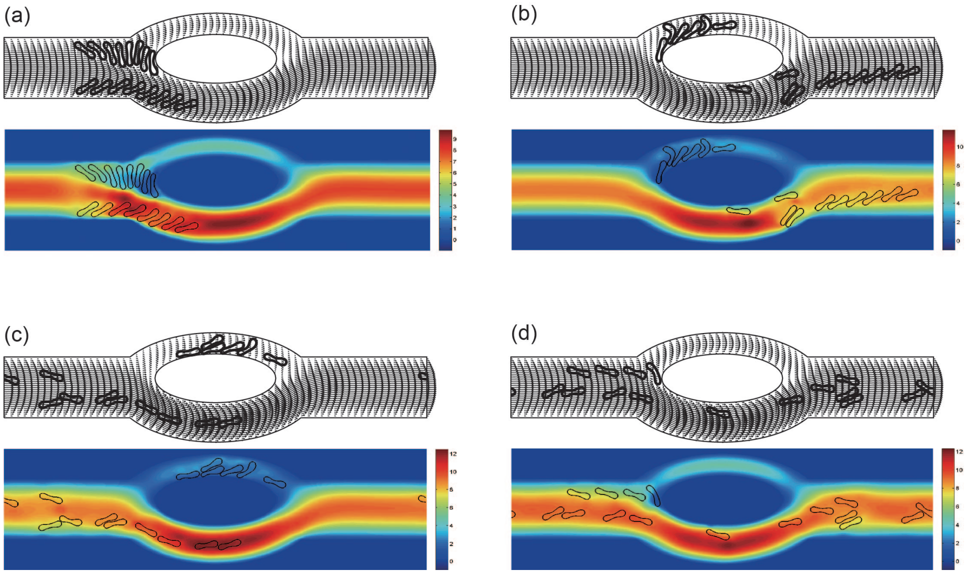

Supplementary Figure S 3: Motion of two files of 16 red blood cells (Hct=6.4 %) in the asymmetric bifurcated microchannel at time instants (a) t = 0.60 ms, (b) t = 1.50 ms, (c) t = 2.40 ms, and (d) t = 5.00 ms. Velocity vectors (upper panels) and axial velocity magnitude contours (cm/s) (lower panels) are presented. The reduced area s^*=0.481. The spring constant of the cell membrane was $k_l = k_b = 3.0 \times 10^{-13}$ Nm.
